# A systematic review of neglected tropical diseases (NTDs) in Myanmar

**DOI:** 10.1371/journal.pntd.0011706

**Published:** 2023-11-01

**Authors:** Myo Maung Maung Swe, Aung Pyae Phyo, Ben S. Cooper, Nicholas J. White, Frank Smithuis, Elizabeth A. Ashley

**Affiliations:** 1 Myanmar Oxford Clinical Research Unit (MOCRU), Yangon, Myanmar; 2 Centre for Tropical Medicine and Global Health, University of Oxford, Oxford, United Kingdom; 3 Shoklo Malaria Research Unit (SMRU), Mahidol-Oxford Tropical Medicine Research Unit, Faculty of Tropical Medicine, Mahidol University, Mae Sot, Thailand; 4 Mahidol-Oxford Tropical Medicine Research Unit (MORU), Faculty of Tropical Medicine, Mahidol University, Bangkok, Thailand; 5 Lao-Oxford-Mahosot Hospital-Wellcome Trust Research Unit, Microbiology laboratory, Mahosot Hospital, Vientiane, Lao People’s Democratic Republic; Consejo Nacional de Investigaciones Cientificas y Tecnicas, Fundación Mundo Sano, ARGENTINA

## Abstract

**Background:**

Neglected tropical diseases (NTDs) affect most impoverished communities in developing countries, like Myanmar in Southeast Asia. NTDs have been understudied and underreported in Myanmar.

**Methods:**

A systematic review of published and grey literature (1900–2023) on neglected tropical diseases (NTDs) in Myanmar was conducted. The literature search included five international databases: PubMed, EMBASE, Ovid Global Health, and Web of Science Core Collection and one national database: the Myanmar Central Biomedical Library (locally published papers and grey literature). The selection criteria included articles with all types of study designs of current or previous infections conducted in humans, that reported NTDs, recognised by WHO, US CDC, and listed in PLoS NTDs. We included melioidosis and rickettsioses which we consider also meet the definition of an NTD.

**Results:**

A total of 5941 records were retrieved and screened, of which, 672 (11%) met the selection criteria and were included in this review. Of the included articles, 449 (65%) were published after 2000 and 369 (55%) were from two regions (Yangon and Mandalay) of Myanmar. Of the included articles, 238 (35%) reported bacterial NTDs, 212 (32%) viral NTDs, 153 (23%) helminth NTDs, 25 (4%) protozoal NTDs and 39 (6%) reported more than one aetiology. Based on reported frequency in descending order, the bacterial NTDs were leprosy, *Escherichia coli* enteritis, salmonellosis, cholera, shigellosis, melioidosis, leptospirosis and rickettsioses; the viral NTDs were dengue, chikungunya and Japanese encephalitis virus (JEV) infection; the protozoal NTDs were amoebiasis, giardiasis and leishmaniasis, and the helminth NTDs were ascariasis, trichuriasis, hookworm disease, filariasis and strongyloidiasis.

**Conclusion:**

This review summarises NTDs reported in Myanmar over the past 100 years. The findings suggest that most NTDs are likely to be under reported, especially from the majority of the country which is far from academic centres. Research capacity building together with strengthening of laboratory systems would lead to better understanding of the true burden of NTDs in Myanmar.

**Trial registration:**

PROSPERO registration ID: CRD42018092627.

## Author summary

Neglected tropical diseases (NTDs) are a group of preventable and treatable diseases common in tropical countries primarily affecting poor rural communities. Myanmar is a tropical country in Southeast Asia in which 70% of the population live in rural areas. It is one of the top ten countries with a high burden of NTDs in term of incidence and prevalence. We conducted a systematic review of all available literature on infectious NTDs in Myanmar to collate all reported NTDs published in international as well as local journals including theses and dissertations. Our review found bacterial and viral NTDs being the most commonly reported NTDs followed by helminthiasis and protozoal NTDs. More than half of the studies were located in two out of fifteen states and regions of Myanmar. This review summarises reported NTDs in Myanmar over 100 years and indicates gaps in knowledge about certain diseases to inform future research directions related to NTDs in Myanmar.

## Introduction

Neglected tropical diseases (NTDs) are a group of preventable and treatable diseases prevailing in tropical countries and which unduly affect the poorest rural communities. The World Health Organization (WHO) lists 20 diseases as NTDs, of which 19 are communicable diseases. The other is snake bite. NTDs cause serious health problems including long term complications and sometimes disfigurement of affected individuals as well as social and economic hardship. The burden of NTDs is tremendous, causing an estimated 200,000 deaths and 19 million disability adjusted life years (DALYs) annually [1]. Approximately 20% of the world’s population, approximately 1.5 billion people, is at risk of NTDs. The Global Burden of Disease (GBD) study estimated that there were 2.3 billion cases of WHO-prioritized NTDs in 2013, with the highest incidence in Southeast Asian countries [2]. NTDs are associated with more disability and disfigurement than deaths. Their collective DALYs burden was comparable to diarrhoeal diseases, HIV/AIDS, tuberculosis and malaria [3]. Despite the devastating burden NTDs impose on global health, research on these diseases has been underfunded compared with HIV/ AIDS, tuberculosis, and malaria [4].

Myanmar (formerly Burma) is one of the least developed countries in Southeast Asia. It has a population of 51 million people, of whom 70% live in rural areas [5,6]. Since 1962, Myanmar has been governed by a military regime, with a brief period of civilian rule between 2011–2020, followed by a coup d’état in early 2021. For several decades, health care expenditure has been consistently low at 0.2 to 0.3% of gross domestic product (GDP) prior to 2011. It increased to 4–6% between 2014 and 2019 [7,8]. This chronic underinvestment in the health sector in Myanmar has resulted in a very weak health care infrastructure, a limited health care workforce, inefficient disease control activities and a high burden of communicable diseases. Infectious diseases still pose a huge burden, with parasitic diseases and other infections causing the most deaths according to 2012 hospital statistics [9]. The global burden of disease (GBD) 2013 study indicates that Myanmar is one of the top ten countries with highest NTD incidence or absolute prevalence [2]. The military coup in 2021 combined with civil war and a collapsed health system will inevitably lead to an increase in diseases of this kind.

With the imposition of economic sanctions combined with neglect under the military regime, the country’s health care system ranked second worst in the world in 2000. There was a slight improvement during the 10 year period of civilian government between 2011 and 2020. Academic research has also declined dramatically, because of severe restriction of professional freedoms [10]. Most of the studies conducted by medical universities and research institutions inside Myanmar were published in local journals, which made them inaccessible to international scholars. For this reason, Myanmar-related studies are typically absent from systematic reviews derived from international databases [11–13]. Because of the lack of investment in healthcare infrastructure for many decades, Myanmar’s laboratory capacity is weak, including inadequate number of microbiologists and laboratory workers.

During World War II, the western alliance troops reported extensive scrub typhus and other rickettsial diseases in northern Myanmar, but reports of such diseases have largely disappeared since then [14,15]. However, a recent serological study in Myanmar indicates a widespread distribution of scrub typhus and other rickettsial diseases [16]. Similarly, melioidosis was also first described in Myanmar in 1912 but went unreported for several decades [17]. Since then, only a few sporadic cases have been reported, except for a recent report of cases from a surveillance study [18]. A recent nationwide study also confirmed that *Burkholderia pseudomallei*, the causative organism, has been isolated in soil in several regions of Myanmar [19]. These points indicate that most of the diseases of regional interest and NTDs in Myanmar are underrecognized or understudied.

Considering these circumstances, a review of these diseases in Myanmar is necessary to gain a better understanding of the current situation. The purpose of this review is to document all the reported NTDs and diseases of regional interest in Myanmar in the past 100 years, considering both peer-reviewed research and the grey literature.

## Methods

This review was conducted in accordance with the Preferred Reporting Items for Systematic Reviews and Meta-Analyses (PRISMA) guideline [20] and the protocol for this review is registered in the international prospective register of systematic reviews (PROSPERO Registration ID: CRD42018092627).

### Diseases (Pathogens) selections for the review

We selected NTDs recognized by the WHO, US CDC, and PLoS Neglected Tropical Diseases to include in this review, with the exception of snake bites because our focus of this review is neglected tropical diseases (NTDs) of infectious origin. We included rickettsial diseases and melioidosis, as diseases of regional interest because they are common in Southeast Asia. However, as other commentators have noted, they are so neglected they are not even recognized as NTDs by the WHO [21].

### Literature search strategy

The database search was carried out in four international databases (PubMed, EMBASE, Ovid Global Health, and Web of Science Core Collection) and one national database (Myanmar Central Biomedical Library) containing grey literature (locally published papers, conference presentations, and dissertations). The last database search was done on 15 May 2023. We used MeSH headings and free text terms without restricting the study design or time period (S1 Table).

### Study selection and full text review

**Fig 1:**
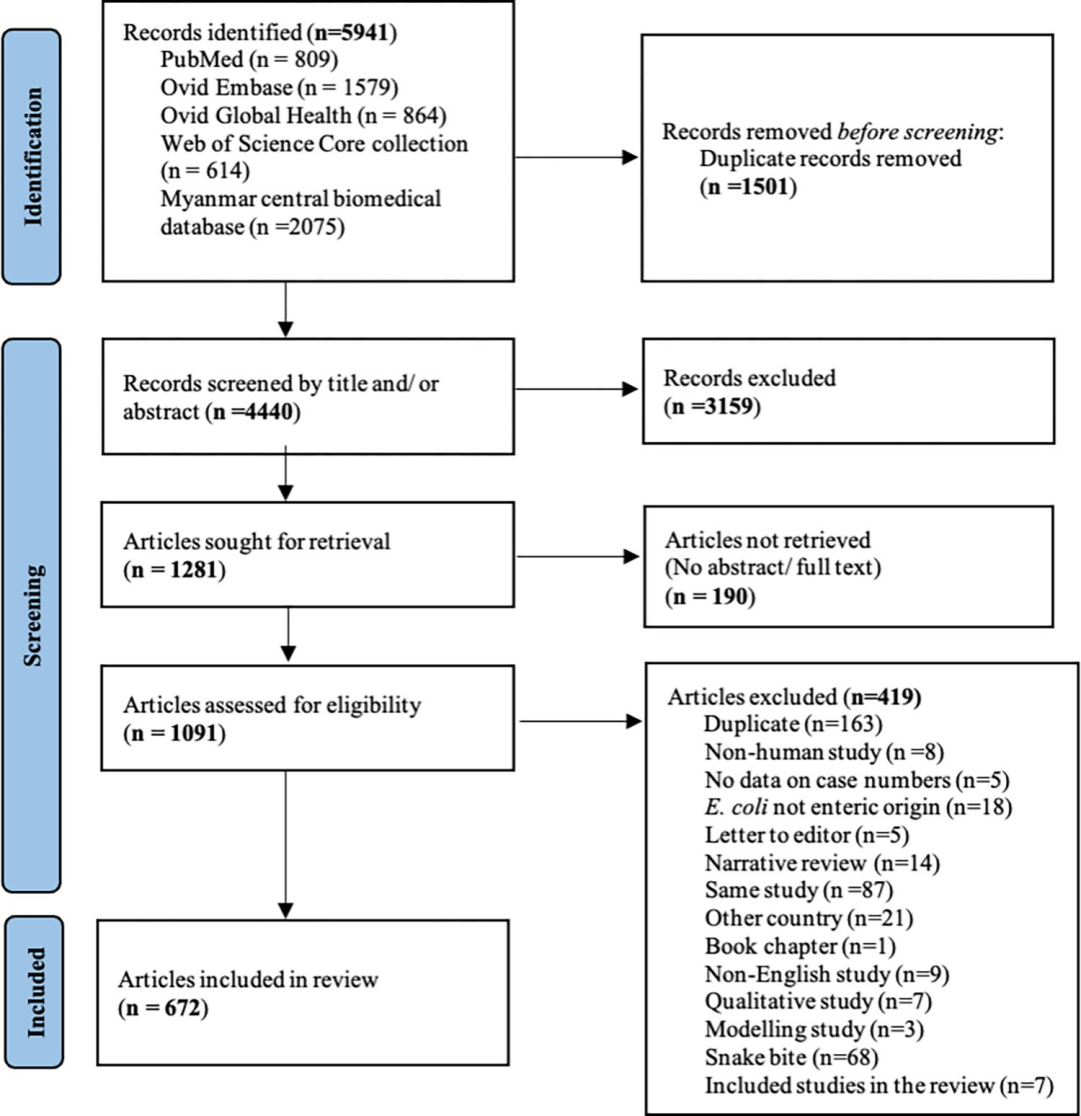
PRISMA flow diagram showing process of article selection.

Initial screening of results from the database search was carried out by two reviewers (MMMS and HNL) who reviewed independently all titles and abstracts. Any discrepancies raised from the screening were resolved by a third reviewer (APP). After screening, we looked at full-text (for published studies) and abstracts (for conference presentations) to assess eligibility against pre-specified selection criteria. Inclusion criteria were observational studies, intervention studies, outbreak reports and investigations, case reports and case series conducted in human population. Editorials, qualitative studies, environmental surveys, non-human studies, and snake bite studies were excluded. For excluded articles the reason for exclusion was recorded. Details of the study selection process are presented in the PRISMA flow diagram (Fig 1).

#### Data extraction

Data from included articles were extracted into a spreadsheet. Extracted variables included the following: year of publication, first author’s name, study period, age group of participants, location of the study, study design, study setting, type of samples, number and name of reported pathogens, methods of diagnosis, number of individuals tested (if reported), number of individuals who tested positive (or) proportion of individuals who tested positive (if reported) for the reported pathogens and title of the study.

#### Included articles

We categorised included articles into (1) case reports and case series, (2) outbreak investigation, (3) surveillance study, (4) observational study (5) intervention study and (7) others.

#### Study population and geographical location

We divided the study population into two groups: children (under 12 years) and adolescents and adults (12 years and above) because children aged 12 years and over are not admitted to paediatric wards but instead, they are admitted to adult wards in Myanmar. In terms of geographic location, articles were grouped into states and regions of Myanmar rather than the exact location.

#### Reported diseases and/ or Pathogens

We categorised reported pathogens and/or diseases into six main groups: viral, bacterial, protozoal, helminths, fungal and ectoparasitic NTDs.

#### Analysis and assessment of risk of bias for diagnosis ascertainment

The included articles varied substantially in terms of study design, population, setting, sample types, diagnostic methods, and information about those being tested. Consequently, the incidence and prevalence of the reported pathogens could not be estimated through meta-analysis. Furthermore, we were not able to assess bias using each domain of currently available tools (RoB-2, QUADAS-2) [22,23]. Thus, we adapted one of the domains of QUADAS-2 (a tool for use in systematic reviews to evaluate the risk of bias and applicability of primary diagnostic accuracy studies). Based on this, we assessed and classified the risk regarding diagnostic ascertainment of the reported pathogens or diseases in the included studies as “high” or “low” risk depending on whether included articles used standard diagnostic methods, recommended by either United States CDC, WHO or literature, to diagnose reported diseases or pathogens (S2 Table). This is because we considered the diagnosis as the largest potential source of bias.

## Results

A total of 5941 articles were identified through the database search. Of these, 672 articles were included in this review (Fig 1). Among them, the earliest was published in 1906 and 65% (449) of them were published after 2000. In terms of the source, 40% (271) of included articles were published in international peer-reviewed journals, 27% (181) were conference papers, 17% (115) were locally published and 11% (75) were theses and dissertations.

**Fig 2:**
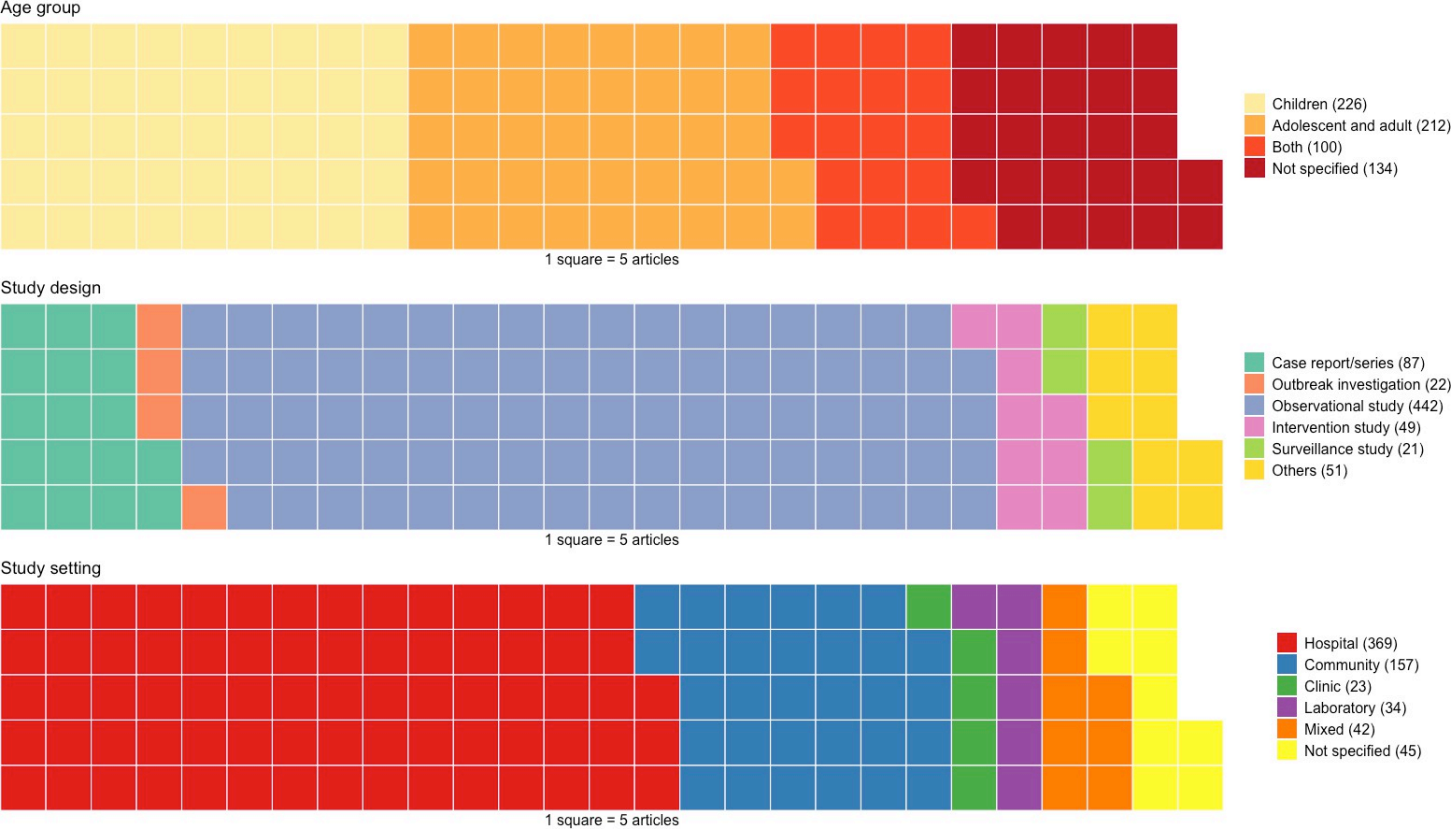
Waffle plots showing distribution of participant age, study design and study setting of included articles.

Regarding study design, more than half (66%, 442) were observational studies followed by 13% (87) case reports or case series, whereas intervention studies accounted for 7% (49), outbreak investigation for 3% (22) and surveillance studies 3% (21) (Fig 2).

### Study population and study settings

In terms of study population, children were the focus of 34% (226) of the included articles, and adolescents and adults were the focus of 32% (212); 15% (100) of articles covered both age groups, and 20% (134) did not specify the age of their subjects (Fig 2). In terms of study setting, 55% (369) were hospital-based studies and 23% (157) were population and community-based studies. The remaining were clinic-based (4%), and laboratory-based (5%); 7% (45) did not specify the type of setting.

### Types of samples

There were a variety of samples collected and analysed in the included articles. The most frequently analysed samples were blood and serum which featured in 254 studies (38%), followed by stool specimens in 171 articles (25%). Cerebrospinal fluid was tested in 6 (1%) articles and 39 (6%) articles analysed more than one type of specimen, while 111 (17%) articles did not specify sample type. The remaining 90 (13%) articles analysed pus, skin smears, wound swabs, and other tissue samples.

### Diagnostic methods

Different diagnostic methods were used in the articles included. They are classified into serology (including immunoassay), culture, molecular, multiple diagnostic methods, others, and not mentioned. Serology was used in 144 (21%) articles, followed by culture in 95 (14%), microscopy in 46 (7%), molecular methods in 39 (6%) while 199 (30%) articles used more than one type of diagnostic method, and 129 (19%) of articles did not mention the diagnostic method.

### Reported aetiology

**Fig 3:**
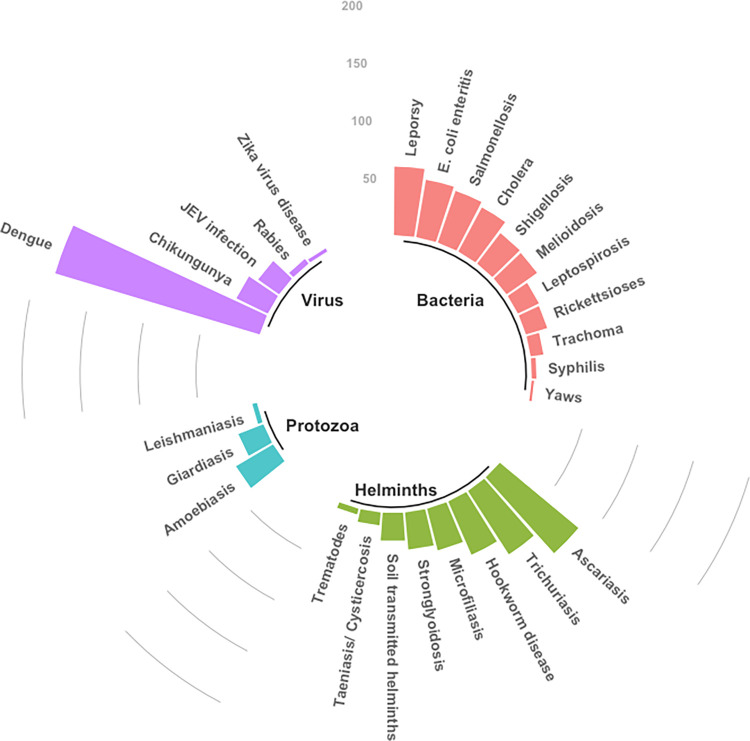
Circular bar plot showing distribution of articles reporting NTDs by aetiology. Four articles that reported deep fungal infection (mycoses) and one article reporting ectoparasitic infection (myiasis) are not shown in the figure for clarity. STH = soil transmitted helminth infections.

Among the 672 included articles, 238 (35%) reported bacterial aetiology, 212 (32%) reported viral aetiology, 153 (23%) reported helminths and 25 (4%) reported protozoal aetiology whereas 39 (6%) reported more than one type of aetiology. In terms of number of reported diseases or pathogens per article, 520 (77%) considered single diseases or pathogens, 137 (20%) reported two to five, while the remaining 15 (2%) reported six to seven diseases or pathogens (Fig 3).

#### Bacterial NTDs

**Fig 4:**
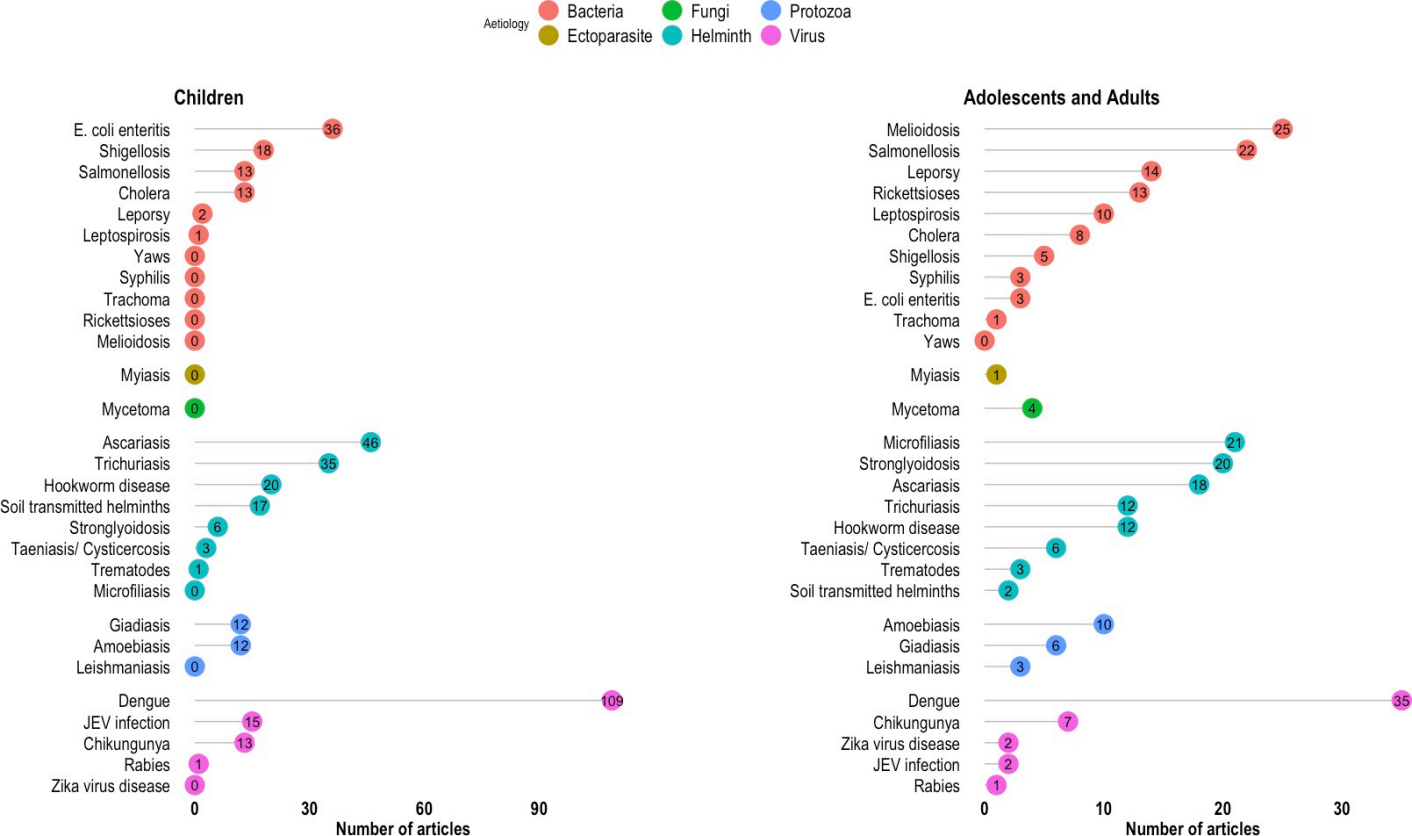
Lollipop plots showing number of articles reporting NTDs stratified by aetiology in children, adolescents and adults.

**Fig 5:**
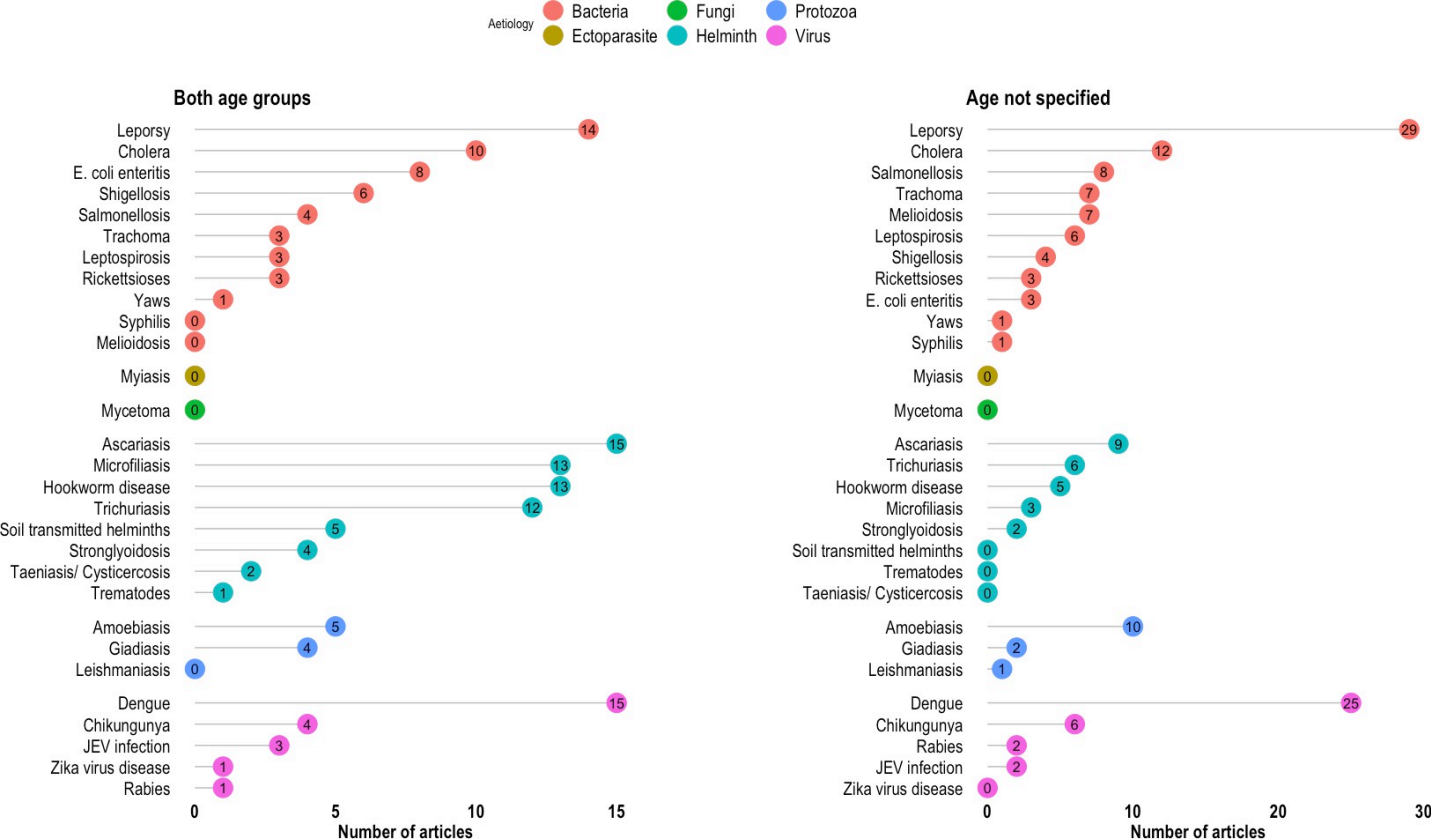
Lollipop plots showing number of articles reporting NTDs stratified by aetiology in both age group and non-specified age group.

Out of 672 articles, at least one bacterial NTD was reported in 320 articles. Among the reported bacterial diseases, enteric bacteria (*Escherichia coli*, *Vibrio cholerae*, *Salmonella* and *Shigella* spp.) were the most frequently reported in 173 articles. NTDs caused by enteric bacteria were reported more frequently in 80 articles reporting findings in children. In contrast, diseases like leprosy, melioidosis, rickettsioses and leptospirosis were reported in 62 articles in the adolescent and adult population (Figs 4 and 5 and S3 Table).

#### Viral NTDs

Out of 672 included articles, 244 reported at least one viral NTD. Arboviral diseases (dengue, Chikungunya, JEV infection and Zika virus disease) were reported in 239 out of 244 articles. The most frequently reported viral pathogen was dengue, which was reported in 184 articles, of which 109 were in children, 35 were in adolescents and adults, 15 were in articles considering both age groups and 25 were in articles where age was not-specified (Figs 4 and 5 and S3 Table). Of the 184 articles that reported dengue infections, seven used clinical diagnosis (WHO dengue classification). Chikungunya and JEV infection were reported in 30 and 22 articles respectively. Rabies virus was reported in only five and Zika virus in three articles.

#### Protozoal NTDs

Three protozoal NTDs were reported in 65 articles included in this review. Amoebiasis was the top reported protozoal NTD, and it was reported in 37 articles followed by Giardiasis in 24 articles. Both were commonly reported in children. Leishmaniasis was reported in four articles (Figs 4 and 5 and S3 Table).

#### Helminthiasis

Helminth NTDs were reported in 312 articles. Ascariasis was the most reported helminthiasis, and it was reported in 88 articles, followed by Trichuriasis and Hookworm infection in 65 and 50 articles. These top three helminth infections were reported more frequently in children compared with adolescents and adults (101 compared with 42 articles). However, Strongyloidiasis was reported more in adolescents and adults than children (20 compared with 6 articles). Microfilariasis was reported in 37 articles, 21 of which considered only adolescents and adults while 13 considered all age groups. Taeniasis, including cysticercosis and trematode infection, was reported in 11 and five articles respectively (Figs 4 and 5 and S3 Table).

#### Fungal NTDs

Deep fungal infection (mycetoma) was reported in four articles (Fig 4 and S3 Table).

#### Ectoparasitic NTDs

Only one article reported a case of myiasis.

#### NTDs reported among returned travellers and Myanmar migrants

Among included articles, 15 reported NTDs in returned travellers, 22 in Myanmar migrants/ refugees in other countries and eight in ex-prisoners-of-war in Myanmar. Strongyloidiasis was reported in all eight articles in ex-prisoners-of-war. Melioidosis and rickettsial diseases were reported in 7 out of 15 articles in returned travellers; the remaining eight articles reported two chikungunya, three salmonellosis, one Zika virus infection, one hookworm infection, and mycetoma.

### Risk of bias regarding diagnostic ascertainment

Of the 672 included articles, the risk of bias in terms of methods used to ascertain diagnosis was considered to be high in 100 (15%), low in 464 (69%) and could not be ascertained in 108 (16%) articles. Most of the articles considered to be of high risk did not report details of diagnostic methods other than vague term such as “serological test” or they did not mention whether the test was done in paired sera.

#### Temporal trend of included articles

In general, the number of articles reporting different NTDs steadily increased over the last 100 years. For articles reporting bacteria and viruses, the number of articles from grey literature which includes locally published articles, conference papers, theses and dissertations is higher than those published in international peer-reviewed journals throughout the years until 2020. There are a few articles reporting protozoal NTDs and most of them were from grey literature. In contrast, most of the articles reporting helminth NTDs were published in peer-reviewed journals. Overall, the total number of articles from grey literature is higher than those from international peer-reviewed journals (S1 Fig).

### Geographic location

**Fig 6:**
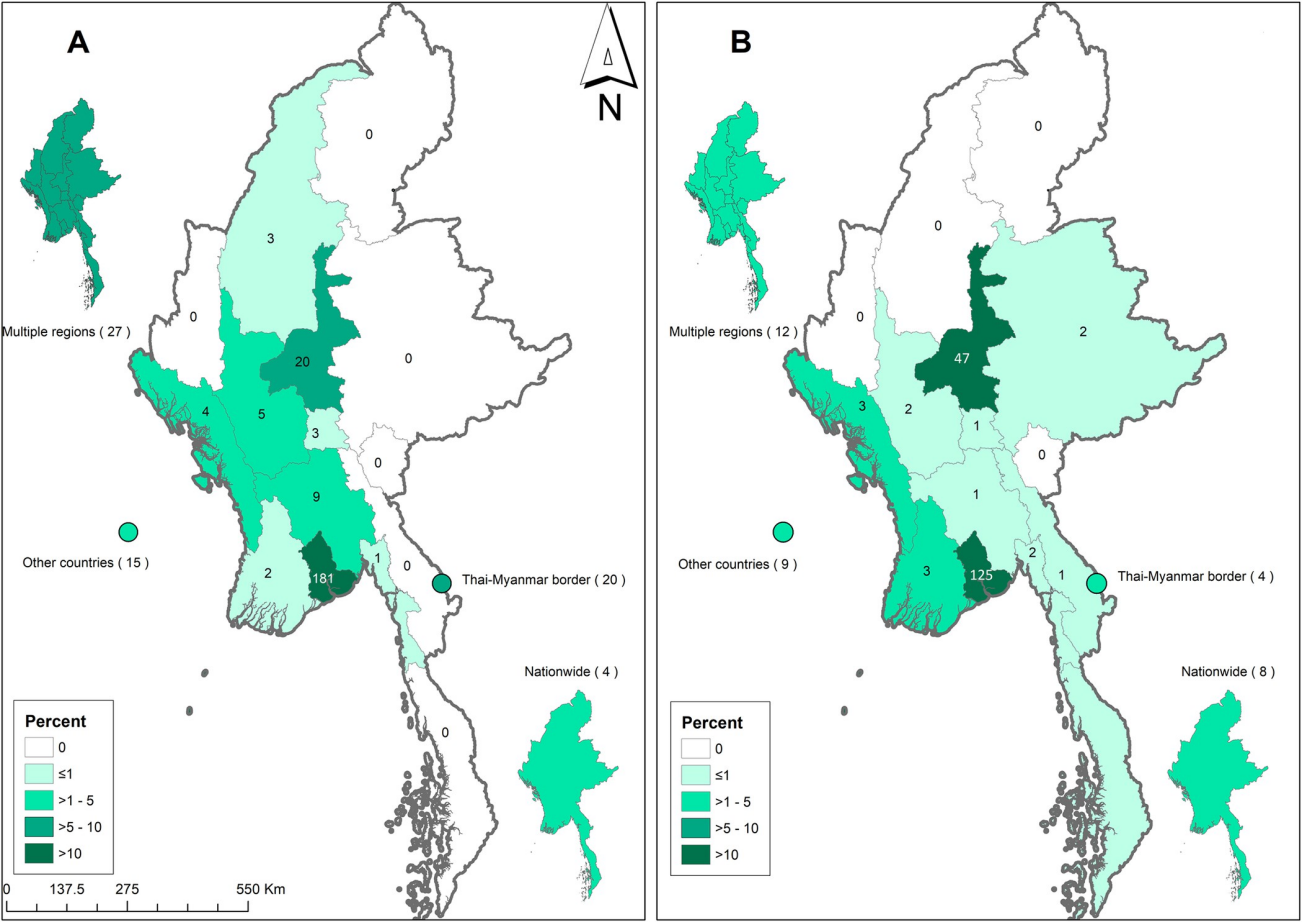
Maps showing location of reported NTDs in the included articles: bacterial NTDs (A) and viral NTDs (B). The number inside each region represents number of pathogens reported in the articles and the colour represents percentage of total articles of corresponding aetiology. Maps were created using ArcGIS version 10.4.1 and the base layer of the map was produced using Geotag (https://geotag.sourceforge.net/).

**Fig 7:**
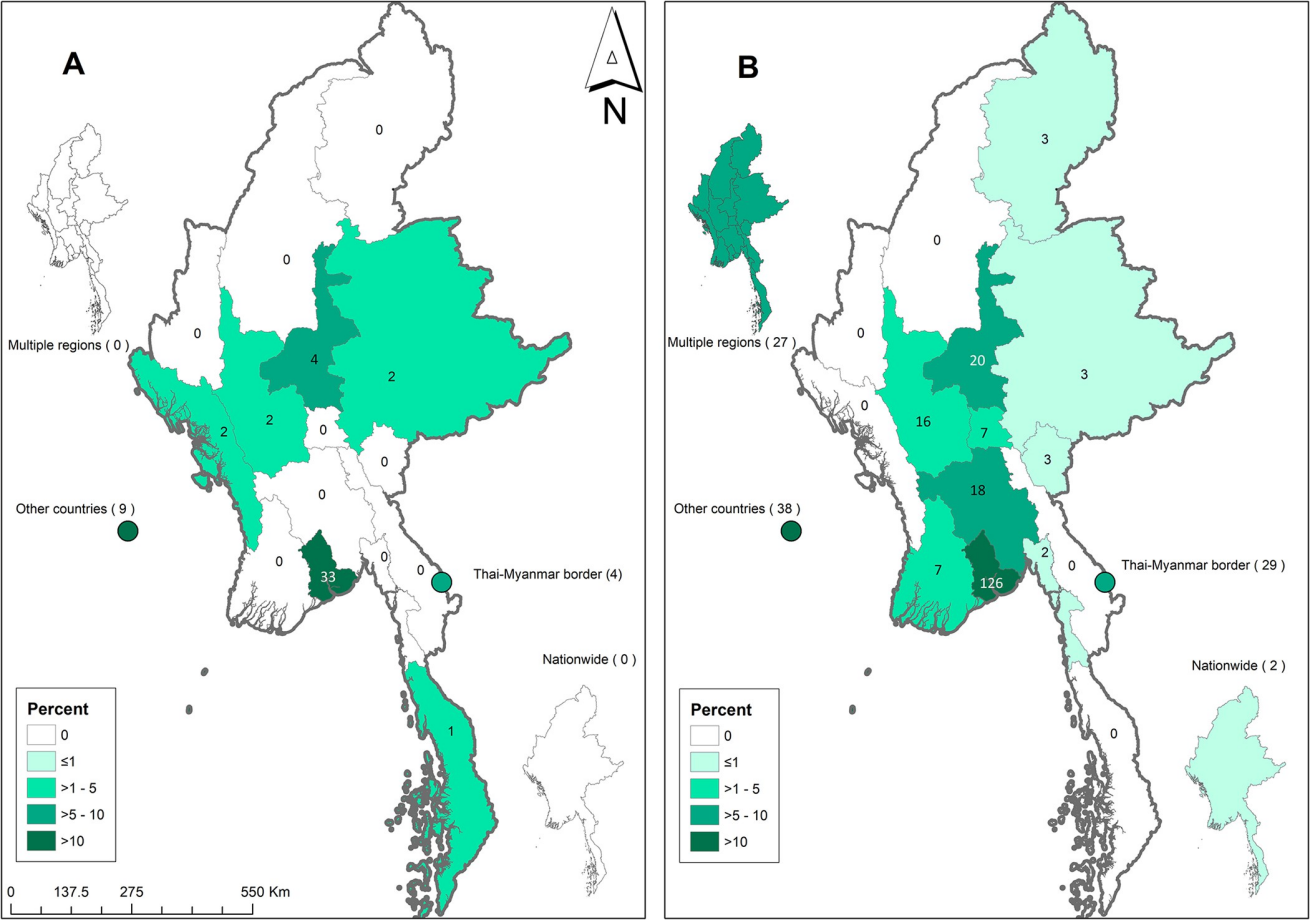
Maps showing location of reported NTDs in the included articles: protozoal NTDs (A) and helminthiasis (B). The number inside each region represents number of pathogens reported in the articles and the colour represents percentage of total articles of corresponding aetiology. Maps were created using ArcGIS version 10.4.1 and the base layer of the map was produced using Geotag (https://geotag.sourceforge.net/).

The majority of articles included were concentrated in two regions of Myanmar: Yangon (45%) and Mandalay (10%). In 29 (4%) articles, the studies were conducted near the Thailand-Myanmar border, while in 13% the location was not specified. A total of 51 (8%) articles were conducted outside Myanmar in travellers returning from Myanmar or in immigrants to other countries from Myanmar. (Figs 6 and 7 and S4 Table).

## Discussion

The purpose of this review was to collate all available information on neglected tropical infectious diseases described in Myanmar in the 100 years until May 2023. We conducted a literature search of four international databases and one national database for locally published articles. We screened 5941 records and included 672 articles in this review.

We present several relevant findings regarding infectious NTDs in this review. In term of aetiology, bacterial NTDs were the most reported, followed by helminthiasis and viral NTDs. NTDs caused by protozoa were reported least frequently. Among bacterial NTDs, enteric diseases that were most reported included *Escherichia coli* enteritis, cholera, salmonellosis, and shigellosis. These enteric pathogens are mainly transmitted through the faecal oral route, and they are associated with poor hygiene practice, contaminated drinking water and low socio-economic conditions. A higher prevalence of these diseases suggests a lack of social development including inaccessibility to clean and safe water in Myanmar. This is supported by recent studies on quality of drinking water in rural and urban regions of Myanmar. A household drinking water survey carried out in a peri-urban region of Myanmar found that 94% of the drinking water samples were contaminated with faecal coliforms and nearly 25% of households did not use any water purification methods for drinking water [24]. Another survey carried out in a rural delta region showed that faecal coliform contamination was detected in all drinking water sources investigated [25]. The studies above suggest the majority of the population residing in both urban and rural Myanmar do not have access to safe drinking water. Improving access to safe drinking water, and improving sanitation and hygiene (WASH) are needed to reduce the burden of these diseases. Ensuring availability of clean water and sanitation to everyone is one of the 17 sustainable development goals adopted by the United Nations Member States in 2015. However, because of the recent coup in Myanmar in early 2021, the situation is likely to deteriorate.

We included melioidosis and rickettsial diseases in this review as they are diseases of regional interest and largely neglected. Several Southeast Asian countries, including Myanmar’s neighbour Thailand, report them frequently and their prevalence in the region is high. For example, Thailand and Laos had severely under-reported melioidosis cases before the disease was actively investigated [26,27]. Melioidosis is now the second most common cause of community-acquired bacteraemia in northeast Thailand, after *E*. *coli*, and the number of melioidosis-related deaths is higher than for common infections like malaria, dengue, measles, and leptospirosis [28]. Although melioidosis was first described in Yangon (Myanmar) in 1911, it largely disappeared from sight for several decades except for a few sporadic reported cases in recent years [17]. A modelling study estimated that the disease burden was still high in Myanmar with an estimated cases of more than 6000 and 3000 deaths annually [29]. In this review, melioidosis was reported in 32 articles most of which considered only adolescents and adults, including three reports in returned travellers and one in a migrant worker. Being an agricultural rice-growing country and with a widespread presence of the bacteria causing melioidosis (*Burkholderia pseudomallei*) in the soil confirmed by a large nationwide soil study, it is likely that melioidosis is under-recognized. A possible explanation could be due to lack of awareness among clinicians as well as lack of familiarity of laboratory staff in isolating the pathogen [19]. In addition, the problem could be compounded by weak laboratory infrastructure together with a shortage of microbiologists.

Scrub typhus, one of the important rickettsioses, occurs mainly in the "tsutsugamushi triangle" area which ranges from northern Japan and Eastern Russia in the north to northern Australia in the south and Pakistan in the west. It causes nearly a million cases each year among an estimated 1 billion people at risk [30,31]. Although Myanmar is located inside the “tsutsugamushi triangle”, rickettsial diseases have not been reported frequently. However, scrub typhus and murine typhus are widespread in Thailand, India, and a leading cause of febrile illness along the Thailand-Myanmar border [32,33]. A recent seroprevalence survey has confirmed widespread distribution of rickettsial diseases in several regions of Myanmar [16]. Given the above circumstances, it is highly likely that rickettsioses are prevalent, but they are being neglected, under-diagnosed and under-recognized in Myanmar. These findings suggest that further studies are necessary to fill the knowledge gap on the burden of rickettsioses as well as melioidosis in Myanmar. The first step in exploring these diseases could be a surveillance study at different sentinel sites based on the results of seroprevalence surveys or environmental studies.

In our review, we identified 244 included articles that reported at least one viral NTD; dengue was the most reported, followed by chikungunya, Japanese encephalitis virus infection, rabies and zika virus disease. These viral NTDs, except rabies, are vector-borne diseases, transmitted through the bite of mosquitoes. These diseases were reported more in children than adolescents and adults. We identified reporting of Zika virus infection in only three articles published between 2018 and 2023. It is notable that outbreaks of these mosquito-borne diseases are associated with urbanization and overcrowding, and success of control measures also depends on community cooperation. Surprisingly, we identified only five articles that reported rabies in this review although the number of reported deaths due to rabies was higher than malaria in Myanmar in 2015 [34]. This finding suggests that rabies in Myanmar is likely to be under-reported.

We found a range of viral and bacterial NTDs that are known to be associated with febrile illness reported in the included articles. Among them, arthropod-borne viral infections were most frequently reported, particularly dengue infection. Among bacterial NTDs associated with febrile illness, salmonellosis was the most reported disease and found in 47 articles. This was followed by melioidosis, rickettsioses and leptospirosis reported in 32, 19 and 20 articles respectively. As reported by neighbouring countries in the region, these diseases might be common aetiologies associated with acute febrile illness in Myanmar, with implications for empirical treatment strategies. For instance, rickettsioses and leptospirosis were reported as common causes of acute febrile illness in Thailand-Myanmar border region and Thailand [32,35].

We identified only three protozoal NTDs: amoebiasis, giardiasis and leishmaniasis in this review. The first two are enteric pathogens transmitted through contaminated water and food. As there is no vaccine for prevention, the primary preventive measure for these diseases is provision of safe water and good hygiene. All four articles that reported leishmaniasis were published after 2004: two in Rakhine state (on the western coast of Myanmar bordering with Bangladesh) and two among Myanmar migrants in Thailand and Malaysia. This suggests *Leishmania* is prevalent among certain populations in Myanmar but simply not being diagnosed and studied. In endemic regions, leishmaniasis has been reported as an important opportunistic infection in people living with HIV [36]. Myanmar has an HIV prevalence of 0.8% [37]. Given that there was no report of leishmaniasis before 2004, it would be of interest to explore disease prevalence in the HIV-infected population.

Helminth NTDs were reported in about a quarter of the included articles. Ascariasis and Trichuriasis are the most reported helminth infections, followed by hookworm infection and filariasis. Soil transmitted helminth (STH) infections are well known to be associated with poor sanitation which is common in impoverished rural populations. Besides eating contaminated vegetables and drinking contaminated water, they can also be transmitted through soil contaminated by eggs from human faeces in places with poor sanitation. WHO recommends administration of annual preventive chemotherapy to target populations which includes pre-school and school-age children as well as reproductive-age women in endemic countries. Myanmar is listed as an STH-endemic country, and preventive chemotherapy has been implemented with high coverage over the last five years [38]. Diagnosis is simple and inexpensive as STH infections are usually diagnosed by microscopic examination of stool. This may explain why a large number of articles related to STH are found in this review.

In terms of location of reported pathogens or diseases in the included articles, there is uneven geographic spread across the 15 regions of Myanmar with more than 50% located in two regions: Yangon and Mandalay. This is likely to reflect the interest of researchers and proximity to research institutions. The findings may also have been influenced by publication bias because accidental findings of cases are more likely to be reported and researched in some regions but not thoroughly investigated elsewhere.

Some NTDs that have been eliminated from neighbouring countries are still prevalent in Myanmar. For instance, Thailand has achieved elimination of lymphatic filariasis as a public health problem since 2017 [39]. However, a number of regions in Myanmar still suffer from persistent transmission of filariasis even after repeated rounds of mass drug administration since the launch of elimination campaign in 2000 [40]. Furthermore, there was a long delay in eliminating other NTDs like trachoma and leprosy, eliminated in 2020 and 2003 respectively, compared to neighbouring countries [41,42].

We found the number of articles reporting NTDs has steadily increased, with grey literature reporting a greater proportion over the last century. This suggests that researchers and academics who are interested in studying specific NTDs in Myanmar could benefit from grey literature as a potential source of information.

This review has several limitations. One of them is heterogeneity of the included articles regarding study designs and diagnostic methods, making them impossible to combine in a meta-analysis. In addition, we were not able to estimate the prevalence or incidence of NTDs given limited information on the denominator and numerator of the study population in the included articles. Moreover, most of the reported NTDs are concentrated in a few regions, making it impossible to interpret them as a true reflection of the distribution across the country. The points above should be taken into account when interpreting the findings. In terms of our risk of bias assessment on ascertainment of diagnosis, 15% of articles were considered to be high risk and 69% to be low risk. Most articles reporting enteric bacterial NTDs, and helminthiasis are in the low-risk category since these NTDs are diagnosed simply by microscopy and culture of stool instead of sophisticated diagnostic methods. In general, we were not able to perform overall risk of bias assessment of the included articles due to unavailability of information for each domain of the currently available bias assessment tools.

Our review also has several strengths. One of these is inclusion of grey literature that is often excluded from systematic reviews. This provides a more comprehensive overview of existing knowledge on neglected tropical diseases in Myanmar. In addition, we described some diseases such as melioidosis and rickettsioses that have disappeared for several decades, but which have been sporadically reported in recent years. These diseases, together with leptospirosis, should be considered as common causes of non-malaria febrile illness and sepsis in Myanmar. With the exception of melioidosis, which requires a prolonged course of specific antibiotics, they can be treated with simple antibiotics such as azithromycin or doxycycline. These findings indicate the necessity to raise awareness of these diseases among clinicians and researchers as well as highlighting the need to gain a better understanding of these diseases in different regions of Myanmar. Furthermore, this review indicates that there is a knowledge gap in the understanding of certain NTDs that are common in the neighbouring countries but less frequently reported in Myanmar.

Last but not least, the findings in this review could be largely shaped by the political situation of Myanmar over several decades. In the past 60 years, there was only a brief period of civilian led government between 2010 and 2021. The military takeover of power in early 2021 and continued low health care budget will likely worsen the fragile health system and increase the burden of NTDs in Myanmar. In an unstable political climate with a collapsing health system, diseases that have been eliminated in recent years may return.

## Conclusion

In conclusion, this systematic review provides an overview of the neglected tropical infectious diseases that had been reported in Myanmar since 1900. It can serve as baseline information and highlights knowledge gaps on certain NTDs and will help to inform the future research direction on NTDs in Myanmar. In addition, the review confirms that certain diseases that are common in Southeast Asia region like melioidosis, leptospirosis and rickettsial diseases also sporadically reported in Myanmar, but they are likely be underreported and unrecognized.

## Supporting information

S1 PRISMA Checklist Preferred Reporting Items for Systematic Reviews and Meta-Analyses (PRISMA). (PDF)

S1 Table Search terms used in the systematic review. (DOCX)

S2 Table Criteria for risk of ascertainment bias assessment. (DOCX)

S3 Table Reported NTDs stratified by aetiology and age groups. (DOCX)

S4 Table Geographic location of reported NTDs in the included articles. (DOCX)

S1 Data List of articles included in the systematic review. (XLS)

S1 Fig Temporal trend of included articles stratified by publication in international peer-reviewed journals (light green) and grey literature (pink) for articles reporting bacteria, virus, protozoa, helminths NTDs and overall. For clarity, four articles reporting mycoses and one reporting myiasis are not shown in the figure.
(TIF)
