# Supplementary material for: A systematic review of neglected tropical diseases (NTDs) in Myanmar
Source: PLoS Negl Trop Dis. 2023 Nov 1;17(11):e0011706. doi: 10.1371/journal.pntd.0011706 (PMC10619876; doi:10.1371/journal.pntd.0011706)
Supplement: S1 Table — (DOCX) [file pntd.0011706.s002.docx]

**Supplementary Table 1. Search terms used in the systematic review**

| **Database** | **Search Terms** |
| --- | --- |
| **PubMed** | **Search date:** 15 May 2023  **Details:** ((((("Myanmar"[Mesh]) OR ((myanmar[Text Word] OR burma[Text Word] OR rangoon[Text Word] OR yangon[Text Word] OR magway[Text Word] OR mandalay[Text Word] OR naypyidaw[Text Word] OR kayah[Text Word] OR ayeyarwady[Text Word] OR bago[Text Word] OR kachin[Text Word] OR sagaing[Text Word] OR kayin[Text Word] OR tanintharyi[Text Word] OR rakhine[Text Word])))))) AND (((((((((((((((((((((((((((((((((((((((((((((((((((((((((((((((((((((((((((((((((((((((((("Buruli Ulcer"[Mesh]) OR "Chagas Disease"[Mesh]) OR "Dengue"[Mesh]) OR "Chikungunya Fever"[Mesh]) OR "Dracunculiasis"[Mesh]) OR "Echinococcosis"[Mesh]) OR "Trematode Infections"[Mesh]) OR "Trypanosomiasis"[Mesh]) OR "Leishmaniasis"[Mesh]) OR "Leprosy"[Mesh]) OR "Elephantiasis, Filarial"[Mesh])) OR "Dermatomycoses"[Mesh]) OR "Onchocerciasis"[Mesh]) OR "Rabies"[Mesh]) OR "Ectoparasitic Infestations"[Mesh]) OR "Trichuriasis"[Mesh]) OR "Strongyloidiasis"[Mesh]) OR "Helminths"[Mesh]) OR "Snake Bites"[Mesh]) OR "Taeniasis"[Mesh]) OR "Trachoma"[Mesh]) OR "Yaws"[Mesh]) OR "Amebiasis"[Mesh]) OR "Balantidiasis"[Mesh]) OR "Giardiasis"[Mesh]) OR "Loiasis"[Mesh]) OR "Toxocariasis"[Mesh]) OR "Larva Migrans"[Mesh]) OR "Encephalitis, Japanese"[Mesh]) OR "Yellow Fever"[Mesh]) OR "Arbovirus Infections"[Mesh]) OR "Hemorrhagic Fevers, Viral"[Mesh]) OR "Bartonella"[Mesh]) OR "Tuberculosis, Bovine"[Mesh]) OR "Cholera"[Mesh]) OR "Shigella"[Mesh]) OR "Enterobacteriaceae"[Mesh]) OR "Leptospirosis"[Mesh]) OR "Borrelia Infections"[Mesh]) OR "Treponemal Infections"[Mesh]) OR "Paracoccidioidomycosis"[Mesh]) OR "Myiasis"[Mesh]) OR "Scrub Typhus"[Mesh]) OR "Typhus, Epidemic Louse-Borne"[Mesh]) OR "Melioidosis"[Mesh])) OR ((buruli ulcer*[Title/Abstract] OR mycobacterium ulcerans[Title/Abstract]))) OR ((chagas[Title/Abstract] OR american trypanosomiasis[Title/Abstract]))) OR dengue[Title/Abstract]) OR chikungunya[Title/Abstract]) OR ((Dracunculiasis[Title/Abstract] OR guinea-worm disease[Title/Abstract] OR guinea-worm infection[Title/Abstract] OR Dracunculosis[Title/Abstract]))) OR ((echinococcosis[Title/Abstract] OR echinococcus infection[Title/Abstract]))) OR ((foodborne trematodiases[Title/Abstract] OR clonorchiasis[Title/Abstract] OR Opisthorchiasis[Title/Abstract] OR Fascioliasis[Title/Abstract] OR Paragonimiasis[Title/Abstract]))) OR ((trypanosomiasis[Title/Abstract] OR sleeping sickness[Title/Abstract]))) OR ((leishmaniasis[Title/Abstract] OR leishmania infection*[Title/Abstract]))) OR ((leprosy[Title/Abstract] OR hansen* disease[Title/Abstract]))) OR ((lymphatic filariasis[Title/Abstract] OR filarial elephantiases[Title/Abstract]))) OR ((mycetoma*[Title/Abstract] OR chromoblastomycosis[Title/Abstract] OR madura foot[Title/Abstract] OR actinomycetoma*[Title/Abstract] OR eumycetoma*[Title/Abstract] OR dermatomycos*[Title/Abstract] OR blastomycos*[Title/Abstract] OR hyalohyphomycos*[Title/Abstract] OR lobomycos*[Title/Abstract] OR sporotrichos*[Title/Abstract] OR tinea[Title/Abstract] OR onychomycos*[Title/Abstract]))) OR ((onchocerciasis[Title/Abstract] OR river blindness[Title/Abstract]))) OR rabies[Title/Abstract]) OR ((scabies[Title/Abstract] OR ectoparasit*[Title/Abstract] OR flea infest*[Title/Abstract] OR tungiasis[Title/Abstract] OR lice infest*[Title/Abstract] OR mite infest*[Title/Abstract] OR trombiculiasis[Title/Abstract] OR myiasis[Title/Abstract] OR hypodermyiasis[Title/Abstract] OR screw worm infest*[Title/Abstract] OR tick infest*[Title/Abstract]))) OR Schistosomias*[Title/Abstract]) OR ((soil-transmitted helminth*[Title/Abstract] OR ascaris[Title/Abstract] OR hookworm*[Title/Abstract] OR Ancylostomatoidea[Title/Abstract] OR whipworm[Title/Abstract] OR Trichuriasis[Title/Abstract] OR Trichuriases[Title/Abstract] OR Strongyloidiasis[Title/Abstract]))) OR ((snakebite envenoming[Title/Abstract] OR snake bit*[Title/Abstract]))) OR ((Taeniasis[Title/Abstract] OR Cysticercosis Taenia[Title/Abstract]))) OR ((Trachoma[Title/Abstract] OR Egyptian Ophthalmia[Title/Abstract]))) OR ((Yaws[Title/Abstract] OR Endemic treponematoses[Title/Abstract] OR Frambesia[Title/Abstract]))) OR ((Amoebiasis[Title/Abstract] OR Amebiases[Title/Abstract] OR Balamuthia Infection*[Title/Abstract]))) OR ((Balantidiasis[Title/Abstract] OR Balantidium Infection*[Title/Abstract]))) OR ((Giardiasis[Title/Abstract] OR Giardia Infection*[Title/Abstract]))) OR ((Loiasis[Title/Abstract] OR Loa Loa infection*[Title/Abstract] OR Loiases[Title/Abstract]))) OR Toxocariasis[Title/Abstract]) OR ((Larva Migrans[Title/Abstract] OR Dew Itch[Title/Abstract]))) OR Japanese encephalitis[Title/Abstract]) OR Yellow fever[Title/Abstract]) OR ((arbovirus infect*[Title/Abstract] OR arboviral infect*[Title/Abstract] OR african horse sickness[Title/Abstract] OR bluetongue[Title/Abstract] OR encephalitis[Title/Abstract] OR west nile fever[Title/Abstract] OR encephalomyelitis[Title/Abstract] OR phlebotomus fever[Title/Abstract] OR rift valley fever[Title/Abstract] OR tick-borne disease*[Title/Abstract] OR african swine fever[Title/Abstract] OR colorado tick fever[Title/Abstract] OR hemorrhagic fever*[Title/Abstract] OR haemorrhagic fever*[Title/Abstract] OR kyasanur forest disease[Title/Abstract] OR nairobi sheep disease[Title/Abstract] OR zika*[Title/Abstract] OR zikv[Title/Abstract]))) OR ((lassa fever[Title/Abstract] OR marburg virus fever[Title/Abstract]))) OR bartonella[Title/Abstract]) OR ((bovine tuberculosis[Title/Abstract] OR bovine tb[Title/Abstract]))) OR cholera[Title/Abstract]) OR ((enteric pathogen*[Title/Abstract] OR enterobacter*[Title/Abstract] OR shigella*[Title/Abstract] OR salmonella*[Title/Abstract] OR e.coli[Title/Abstract] OR e coli[Title/Abstract] OR escherichia[Title/Abstract]))) OR ((leptospiros*[Title/Abstract] OR stuttgart disease[Title/Abstract] OR leptospira infection*[Title/Abstract] OR weil disease[Title/Abstract]))) OR ((relapsing fever*[Title/Abstract] OR borrelia infect*[Title/Abstract]))) OR ((treponematos*[Title/Abstract] OR bejel[Title/Abstract] OR pinta[Title/Abstract] OR syphili*[Title/Abstract] OR treponemal[Title/Abstract]))) OR Paracoccidioidomycos*[Title/Abstract]) OR ((Blastomyces[Title/Abstract] OR brasiliensis Infection[Title/Abstract]))) OR ((Myiasis[Title/Abstract] OR Maggot Infest*[Title/Abstract]))) OR ((typhus[Title/Abstract] OR brill* disease[Title/Abstract] OR tsutsugamushi[Title/Abstract] OR jail fever[Title/Abstract]))) OR ((Melioidos*[Title/Abstract] OR Burkholderia pseudomallei Infection[Title/Abstract]))) |
| **Embase** | **Search date:** 15 May 2023  **Details:**  1 Myanmar/  2 (myanmar or burma or rangoon or yangon or magway or mandalay or naypyidaw or kayah or ayeyarwady or bago or kachin or sagaing or kayin or tanintharyi or rakhine).tw.  3 1 or 2  4 Buruli ulcer/  5 exp dengue/  6 chikungunya/ or chikungunya virus/  7 dracunculiasis/  8 exp echinococcosis/  9 exp trematodiasis/  10 exp trypanosomiasis/  11 exp leishmaniasis/  12 exp leprosy/  13 exp lymphatic filariasis/  14 exp mycetoma/  15 exp dermatomycosis/  16 exp onchocerciasis/  17 rabies/  18 exp ectoparasitosis/  19 exp helminth/  20 exp strongyloidiasis/  21 snakebite/  22 exp taeniasis/  23 trachoma/  24 exp treponematosis/  25 exp amebiasis/  26 balantidiasis/  27 giardiasis/  28 loiasis/  29 toxocariasis/  30 exp larva migrans/  31 Arbovirus/  32 exp RNA virus infection/  33 virus hemorrhagic fever/  34 bartonella/  35 bovine tuberculosis/  36 cholera/  37 exp Enterobacteriaceae/  38 leptospirosis/  39 exp Borrelia infection/  40 South American blastomycosis/  41 exp myiasis/  42 exp typhus/  43 melioidosis/  44 ("buruli ulcer*" or "mycobacterium ulcerans" or chagas or "american trypanosomiasis" or dengue or chikungunya or dracunculiasis or "guinea-worm disease" or "guinea-worm infection*" or dracunculosis or echinococcosis or echinococcus or "foodborne trematodiases" or clonorchiasis or opisthorchiasis).ti,ab.  45 (Fascioliasis or paragonimiasis or trypanosomiasis or "sleeping sickness" or leishmaniasis or "leishmania infection*" or leprosy or "hansen* disease" or "lymphatic filariasis" or "filarial elephantiases" or mycetoma* or chromoblastomycosis or "madura foot" or actinomycetoma* or eumycetoma* or dermatomycos* or blastomycos* or hyalohyphomycos* or lobomycos* or sporotrichos* or tinea or onychomycos* or onchocerciasis or "river blindness" or rabies or scabies or ectoparasit* or "flea infest*" or tungiasis or "lice infest*" or "mite infest*" or trombiculiasis).ti,ab.  46 (myiasis or hypodermyiasis or "screw worm infest*" or "tick infest*" or schistosomias* or "soil-transmitted helminth*" or ascaris or hookworm* or Ancylostomatoidea or whipworm or trichuriasis or trichuriases or strongyloidiasis or snakebite* or "snake bit*" or taeniasis or "cysticercosis taenia" or trachoma or "egyptian ophthalmia" or yaws or "endemic treponematoses" or frambesia or amoebias* or amebias* or "balamuthia infection*" or balantidiasis or "balantidium infection*" or giardiasis or "giardia infection*" or loiasis or "loa loa infection*" or loiases or toxocariasis or "larva migrans" or "dew itch" or "japanese encephalitis" or "yellow fever" or "arbovirus infect*" or "arboviral infect*" or "african horse sickness" or bluetongue or encephalitis or "west nile fever" or encephalomyelitis or "phlebotomus fever" or "rift valley fever" or "tick-borne disease*" or "african swine fever" or "colorado tick fever" or "hemorrhagic fever*" or "haemorrhagic fever*" or "kyasanur forest disease" or "nairobi sheep disease" or zika*).ti,ab. (137522)  47 (zikv or "lassa fever" or "marburg virus fever" or bartonella or "bovine tuberculosis" or "bovine tb" or cholera or "enteric pathogen*" or enterobacter* or shigella* or salmonella* or "e.coli" or "e coli" or escherichia or leptospiros* or "stuttgart disease" or "leptospira infection*" or "weil disease" or "relapsing fever*" or "borrelia infect*" or treponematos* or bejel or pinta or syphili* or treponemal or Paracoccidioidomycos* or blastomyces or "brasiliensis infection" or myiasis or "maggot infest*" or typhus or "brill* disease" or tutsugamushi or "jail fever" or melioidos* or burkholderia).ti,ab.  48 4 or 5 or 6 or 7 or 8 or 9 or 10 or 11 or 12 or 13 or 14 or 15 or 16 or 17 or 18 or 19 or 20 or 21 or 22 or 23 or 24 or 25 or 26 or 27 or 28 or 29 or 30 or 31 or 32 or 33 or 34 or 35 or 36 or 37 or 38 or 39 or 40 or 41 or 42 or 43 or 44 or 45 or 46 or 47  49 3 and 48 |
| **Global Health** | 1 Myanmar/  2 (myanmar or burma or rangoon or yangon or magway or mandalay or naypyidaw or kayah or ayeyarwady or bago or kachin or sagaing or kayin or tanintharyi or rakhine).tw.  3 1 or 2  4 Buruli ulcer/  5 exp dengue/  6 chikungunya/ or chikungunya virus/  7 dracunculiasis/  8 exp echinococcosis/  9 exp foodborne diseases/  10 exp trypanosomiasis/  11 exp leishmaniasis/  12 exp leprosy/  13 exp lymphatic filariasis/  14 exp mycetoma/  15 exp dermatomycosis/  16 exp onchocerciasis/  17 rabies/  18 exp ectoparasites/  19 exp helminths/  20 exp strongyloidiasis/  21 snake venom/  22 exp taeniasis/  23 trachoma/  24 exp treponematosis/  25 amoebiasis/  26 balantidiasis/  27 giardiasis/  28 loiasis/  29 toxocariasis/  30 exp larva migrans/  31 arboviruses/  32 exp viral haemorrhagic fevers/  33 bartonella/  34 exp mycobacterium bovis/  35 cholera/  36 exp Enterobacteriaceae/  37 leptospirosis/  38 exp borrelia/  39 South American blastomycosis/  40 exp myiasis/  41 exp typhus/  42 melioidosis/  43 ("buruli ulcer*" or "mycobacterium ulcerans" or chagas or "american trypanosomiasis" or dengue or chikungunya or dracunculiasis or "guinea-worm disease" or "guinea-worm infection*" or dracunculosis or echinococcosis or echinococcus or "foodborne trematodiases" or clonorchiasis or opisthorchiasis).ti,ab.  44 (Fascioliasis or paragonimiasis or trypanosomiasis or "sleeping sickness" or leishmaniasis or "leishmania infection*" or leprosy or "hansen* disease" or "lymphatic filariasis" or "filarial elephantiases" or mycetoma* or chromoblastomycosis or "madura foot" or actinomycetoma* or eumycetoma* or dermatomycos* or blastomycos* or hyalohyphomycos* or lobomycos* or sporotrichos* or tinea or onychomycos* or onchocerciasis or "river blindness" or rabies or scabies or ectoparasit* or "flea infest*" or tungiasis or "lice infest*" or "mite infest*" or trombiculiasis).ti,ab.  45 (myiasis or hypodermyiasis or "screw worm infest*" or "tick infest*" or schistosomias* or "soil-transmitted helminth*" or ascaris or hookworm* or Ancylostomatoidea or whipworm or trichuriasis or trichuriases or strongyloidiasis or snakebite* or "snake bit*" or taeniasis or "cysticercosis taenia" or trachoma or "egyptian ophthalmia" or yaws or "endemic treponematoses" or frambesia or amoebias* or amebias* or "balamuthia infection*" or balantidiasis or "balantidium infection*" or giardiasis or "giardia infection*" or loiasis or "loa loa infection*" or loiases or toxocariasis or "larva migrans" or "dew itch" or "japanese encephalitis" or "yellow fever" or "arbovirus infect*" or "arboviral infect*" or "african horse sickness" or bluetongue or encephalitis or "west nile fever" or encephalomyelitis or "phlebotomus fever" or "rift valley fever" or "tick-borne disease*" or "african swine fever" or "colorado tick fever" or "hemorrhagic fever*" or "haemorrhagic fever*" or "kyasanur forest disease" or "nairobi sheep disease" or zika*).ti,ab.  46 (zikv or "lassa fever" or "marburg virus fever" or bartonella or "bovine tuberculosis" or "bovine tb" or cholera or "enteric pathogen*" or enterobacter* or shigella* or salmonella* or "e.coli" or "e coli" or escherichia or leptospiros* or "stuttgart disease" or "leptospira infection*" or "weil disease" or "relapsing fever*" or "borrelia infect*" or treponematos* or bejel or pinta or syphili* or treponemal or Paracoccidioidomycos* or blastomyces or "brasiliensis infection" or myiasis or "maggot infest*" or typhus or "brill* disease" or tutsugamushi or "jail fever" or melioidos* or burkholderia).ti,ab.  47 4 or 5 or 6 or 7 or 8 or 9 or 10 or 11 or 12 or 13 or 14 or 15 or 16 or 17 or 18 or 19 or 20 or 21 or 22 or 23 or 24 or 25 or 26 or 27 or 28 or 29 or 30 or 31 or 32 or 33 or 34 or 35 or 36 or 37 or 38 or 39 or 40 or 41 or 42 or 43 or 44 or 45 or 46  48 3 and 47 |
| **Web of science** | # 1 10,377 TOPIC: (myanmar or burma or rangoon or yangon or magway or mandalay or naypyidaw or kayah or ayeyarwady or bago or kachin or sagaing or kayin or tanintharyi or rakhine)  Indexes=SCI-EXPANDED, SSCI, A&HCI, CPCI-S, CPCI-SSH, BKCI-S, BKCI-SSH, ESCI, CCR-EXPANDED, IC Timespan=All years  # 2 54,446 TOPIC: ("buruli ulcer*" or "mycobacterium ulcerans" or chagas or "american trypanosomiasis" or dengue or chikungunya or dracunculiasis or "guinea-worm disease" or "guinea-worm infection*" or dracunculosis or echinococcosis or echinococcus or "foodborne trematodiases" or clonorchiasis or opisthorchiasis)  Indexes=SCI-EXPANDED, SSCI, A&HCI, CPCI-S, CPCI-SSH, BKCI-S, BKCI-SSH, ESCI, CCR-EXPANDED, IC Timespan=All years  # 3 102,873 TOPIC: (Fascioliasis or paragonimiasis or trypanosomiasis or "sleeping sickness" or leishmaniasis or "leishmania infection*" or leprosy or "hansen* disease" or "lymphatic filariasis" or "filarial elephantiases" or mycetoma* or chromoblastomycosis or "madura foot" or actinomycetoma* or eumycetoma* or dermatomycos* or blastomycos* or hyalohyphomycos* or lobomycos* or sporotrichos* or tinea or onychomycos* or onchocerciasis or "river blindness" or rabies or scabies or ectoparasit* or "flea infest*" or tungiasis or "lice infest*" or "mite infest*" or trombiculiasis)  Indexes=SCI-EXPANDED, SSCI, A&HCI, CPCI-S, CPCI-SSH, BKCI-S, BKCI-SSH, ESCI, CCR-EXPANDED, IC Timespan=All years  # 4 168,137 TOPIC: (myiasis or hypodermyiasis or "screw worm infest*" or "tick infest*" or schistosomias* or "soil-transmitted helminth*" or ascaris or hookworm* or Ancylostomatoidea or whipworm or trichuriasis or trichuriases or strongyloidiasis or snakebite* or "snake bit*" or taeniasis or "cysticercosis taenia" or trachoma or "egyptian ophthalmia" or yaws or "endemic treponematoses" or frambesia or amoebias* or amebias* or "balamuthia infection*" or balantidiasis or "balantidium infection*" or giardiasis or "giardia infection*" or loiasis or "loa loa infection*" or loiases or toxocariasis or "larva migrans" or "dew itch" or "japanese encephalitis" or "yellow fever" or "arbovirus infect*" or "arboviral infect*" or "african horse sickness" or bluetongue or encephalitis or "west nile fever" or encephalomyelitis or "phlebotomus fever" or "rift valley fever" or "tick-borne disease*" or "african swine fever" or "colorado tick fever" or "hemorrhagic fever*" or "haemorrhagic fever*" or "kyasanur forest disease" or "nairobi sheep disease" or zika*)  Indexes=SCI-EXPANDED, SSCI, A&HCI, CPCI-S, CPCI-SSH, BKCI-S, BKCI-SSH, ESCI, CCR-EXPANDED, IC Timespan=All years  # 5 654,594 TOPIC: (zikv or "lassa fever" or "marburg virus fever" or bartonella or "bovine tuberculosis" or "bovine tb" or cholera or "enteric pathogen*" or enterobacter* or shigella* or salmonella* or "e.coli" or "e coli" or escherichia or leptospiros* or "stuttgart disease" or "leptospira infection*" or "weil disease" or "relapsing fever*" or "borrelia infect*" or treponematos* or bejel or pinta or syphili* or treponemal or Paracoccidioidomycos* or blastomyces or "brasiliensis infection" or myiasis or "maggot infest*" or typhus or "brill* disease" or tutsugamushi or "jail fever" or melioidos* or burkholderia)  Indexes=SCI-EXPANDED, SSCI, A&HCI, CPCI-S, CPCI-SSH, BKCI-S, BKCI-SSH, ESCI, CCR-EXPANDED, IC Timespan=All years  # 6 949,413 #5 OR #4 OR #3 OR #2  Indexes=SCI-EXPANDED, SSCI, A&HCI, CPCI-S, CPCI-SSH, BKCI-S, BKCI-SSH, ESCI, CCR-EXPANDED, IC Timespan=All years  # 7 382 #6 AND #1  Indexes=SCI-EXPANDED, SSCI, A&HCI, CPCI-S, CPCI-SSH, BKCI-S, BKCI-SSH, ESCI, CCR-EXPANDED, IC Timespan=All years |
| **Central Biomed Library (Myanmar database)** | 1. Buruli ulcer  2. Mycobact  3. Chagas disease  4. Trypnosomiasis  4. Dengue  5. Chikungunya  6. Arbovir  7. Dracunculiasis  8. guinea-worm disease  9. Echinococcosis  10. Trematodiases  11. Clonorchiasis  12. Opisthorchiasis  13. Fascioliasis  14. Paragonimiasis  15. Paragonimus  15. Trematode  16. Trematode worms  17. Worms  18. Flukes  19. Parasit  20. Trypanosomiasis  21. Sleeping sickness  22. Leishmaniasis  24. Leprae  25. Lepr  26. Filaria  27. Wuchereria  28. Mycetoma  29. Chromoblastomycosis  30. Mycoses  31. Onchocerciasis  32. River blindness  33. Rabies  34. Scabies  35. Sarcoptes scabiei  36. Ectoparasites  37. Schistosomiasis  38. Helminth  39. Ascaris  40. Hookworm  41. Whipworm  42. Trichuriasis  43. Strongyloid  44. Snake bite  45. Taenia  46. Cysticercosis  47. Trachoma  48. Chlamydia  49. Yaws  50. Treponema  51. Treponematoses  52. Amoebiasis  53. Entamoeba  54. Dysentery  55. Balantidiasis  56. Balantidium  57. Giardia  58. Diarrhoea  59. Loiasis  60. Toxocariasis  61. Larva Migrans  62. Encephalitis  63. Fever  64. Rift Valley fever  65. Zoonoses  66. Haemorrhagic fever  67. Bartonella  68. Tuberculosis  69. Cholera  70. Vibrio  71. Shigella  72. Salmonella  73. Escherichia  74. Enterobacteriaceae  75. Lepto  76. Borrelia  77. Treponematoses  78. Eumycetoma  79. Paracoccidioidomycosis  80. Myiasis  81. Typhus  82. Typhoid  83. Rickettsia  84. Melioid  85. Burkholderia |
