# Supplementary material for: A systematic review of neglected tropical diseases (NTDs) in Myanmar
Source: PLoS Negl Trop Dis. 2023 Nov 1;17(11):e0011706. doi: 10.1371/journal.pntd.0011706 (PMC10619876; doi:10.1371/journal.pntd.0011706)
Supplement: S2 Table — (DOCX) [file pntd.0011706.s003.docx]

**Supplementary Table 2. Criteria for risk of ascertainment bias assessment**

| **Bacteria** | **Low risk** | **High risk** | **Reference** |
| --- | --- | --- | --- |
| *Escherichia coli* enteritis | Isolation of the pathogen by culture of clinical specimen (or) detection of nucleic acid by molecular method (PCR) | microscopy | <https://www.cdc.gov/ecoli/clinicians.html> |
| Cholera | Identification of the pathogen by direct microscopy (dark field microscopy) (or) culture of stools (or) detection of genetic material by PCR | Rapid Antigen test | <https://www.cdc.gov/cholera/diagnosis.html> |
| Salmonellosis | Isolation of the pathogen by culture of clinical specimens (or) PCR | Serology (e.g., Widal test) | <https://www.cdc.gov/typhoid-fever/health-professional.html> |
| Shigellosis | Isolation of shigella from faeces/ rectal swabs (or) detection of genetic material/ virulent gene by PCR | routine microscopy (*not specific for shigella species) | <https://emedicine.medscape.com/article/968773-workup> |
| Leprosy | clinical examination with or without detection of acid-fast bacilli in slit skin smear/ skin or nerve biopsy (or) Isolation of the pathogen by culture (or) PCR | stand-alone serology (anti-PGL-I and anti-LID-1 antibody titres by ELISA and by the NDO-LID rapid test) | <https://apps.who.int/iris/bitstream/handle/10665/274127/9789290226383-eng.pdf> |
| Melioidosis | Isolation and identification of the pathogen by culture of clinical specimens (or) detection of genetic material by PCR (or) Rapid detection of the organism in the specimen by IFA | Serology (Indirect Haemagglutination test/ ELISA) | <https://wwwnc.cdc.gov/eid/article/21/2/14-1045_article> |
| Rickettsiosis | Isolation by culture (or) detection of genetic material by PCR/ loop amplification (or) **paired serology** by IFA/ ELISA (≥ 4-fold increase in antibody titer between two consecutive samples) | Weil-Felix test, rapid Antigen test | <https://www.ncbi.nlm.nih.gov/pmc/articles/PMC2829893/> |
| Leptospirosis | Paired serology (≥ 4-fold increase in antibody titre) by microscopic agglutination test (MAT) / a single high MAT titer > 400 (or) PCR | positive MAT titre < 1/400 (or) ELISA (or) dark field microscopy (or) IgM-specific serologic screening test |  |
| Trachoma | Clinical diagnosis in endemic region |  |  |
| Syphilis | Direct detection methods (i.e. dark-field microscopy, direct fluorescent antibody test and nucleic acid amplification test) (or) positive serological tests for both non-treponemal (VDRL/ PRP) and treponemal (TPHA/ TPPA) tests | either non-treponemal test or treponemal test performed | <https://www.who.int/reproductivehealth/publications/rtis/syphilis-ANC-screenandtreat-guidelines/en/> |
| Yaws | Dual Path Platform Syphilis Screen and Confirm assay, detection of DNA in skin lesion by PCR | Treponema pallidum particle agglutination (TPPA) and rapid plasma reagin (RPR) (* these tests cannot differentiate yaws from syphilis) | <https://www.who.int/news-room/fact-sheets/detail/yaws> |
|  |  |  |  |
| **Virus** | **Low risk (Standard Laboratory case definition)** | **High risk** |  |
| Dengue infection | Viral isolation (or) Detection of DENV nucleic acid (or) NS1 Ag in clinical specimens (or) IgM/ IgG Anti-DENV seroconversion in acute and convalescent serums (or) > 4-fold rise in titres in serum specimens collected 2 weeks apart. | IgM/ IgG anti-DENV detection in a single serum sample | <https://www.cdc.gov/dengue/healthcare-providers/diagnosis.html> |
| Chikungunya infection | Detection of the viral nucleic acid by PCR (or) detection of chikungunya IgM antibody confirmed with neutralizing antibodies | detection of chikungunya IgM antibody | <https://www.cdc.gov/chikungunya/hc/diagnostic.html> |
| Japanese encephalitis virus infection | Detection of viral nucleic acid by PCR (or) detection of JE virus IgM antibody in CSF and serum confirmed with neutralizing antibody testing | JE virus IgM antibody in a single CSF/ serum specimen (due to cross reactivity with other flavivirus) | <https://www.cdc.gov/japaneseencephalitis/healthcareproviders/healthcareproviders-diagnostic.html#:~:text=Laboratory%20diagnosis%20of%20JE%20is,longer%20persistence%20has%20been%20documented.> |
| Rabies infection | Viral isolation (or) detection of viral nucleic acid by PCR (or) rabies antigen in clinical specimens (human) (or) Detection of viral antigen in the tissues of animals. (or) Clinical pictures that are compatible with rabies with preceding history of dog bite. |  | <https://www.cdc.gov/rabies/diagnosis/animals-humans.html#:~:text=Diagnosis%20in%20humans&text=Saliva%20can%20be%20tested%20by,the%20base%20of%20hair%20follicles.> |
| Zika virus infection | Detection of virus nucleic acid by PCR in the clinical specimens (or) Plaque reduction neutralization test (quantitative assays that measure virus-specific neutralizing antibody titres) | Detection of Zika virus IgM antibody (due to cross-reactivity with other flavivirus). | <https://www.cdc.gov/zika/laboratories/types-of-tests.html> |
|  |  |  |  |
| **Protozoa** | **Low risk** | **High risk** |  |
| Amoebiasis | Conventional/ real-time PCR (or) Antigen (or) Antibody detection (ELISA/ IHA/ IFA) | Identification of the parasites (cysts/ trophozoites) in clinical specimens by microscopy | <https://www.cdc.gov/dpdx/amebiasis/index.html> |
| Giardiasis | Microscopy with direct fluorescent antibody testing (DFA) (or) PCR test (or) Ag detection by ELISA |  | <https://www.cdc.gov/parasites/giardia/medical-professionals.html> |
| Leishmaniasis | Detection of Leishmania parasites (DNA) by microscopy/culture of tissue specimens or detection of parasite DNA by PCR | Serology (IFA/ ELISA) *most serology assays do not reliably distinguish active and quiescent infections | <https://www.cdc.gov/parasites/leishmaniasis/health_professionals/index.html> |
|  |  |  |  |
| **Helminth** | **Low risk** | **High risk** |  |
| Ascariasis | Detection of eggs/ lava by microscopy-based methods or identification of adult worm (or) molecular methods (including PCR) |  | <https://www.ncbi.nlm.nih.gov/pmc/articles/PMC5652060/> |
| Hookworm infection | Detection of eggs/ lava by microscopy-based methods or identification of adult worm (or) molecular methods (including PCR) |  | <https://www.ncbi.nlm.nih.gov/pmc/articles/PMC5652060/> |
| Strongyloidiasis | Detection of eggs/ lava by microscopy-based methods or identification of adult worm (or) molecular methods (including PCR) |  | <https://www.ncbi.nlm.nih.gov/pmc/articles/PMC5652060/> |
| Microfilariasis | blood smear examination by microscopy (or) Antigen/ Antibody detection by immunoassays (or) Antigen detection by rapid test |  | <https://www.cdc.gov/dpdx/lymphaticFilariasis/index.html> |
| Trichuriasis | Detection of eggs/ lava by microscopy-based methods |  | <https://www.cdc.gov/dpdx/trichuriasis/> |
| Taeniasis | Detection of eggs/ lava by microscopy-based methods or identification of adult worm (or) molecular methods (including PCR), serology test (Ag/ Ab) detection) , Imaging (CT/ MRI) for cysticercosis |  | <https://www.cdc.gov/parasites/taeniasis/health_professionals/index.html> |
| Trematodes infection | detection of ova in stool/ urine samples by microscopy (or) PCR | Serological testing (Antibody to adult worm) | [https://www.cdc.gov/parasites/schistosomiasis/health_professionals/index.html#dx](https://www.cdc.gov/parasites/schistosomiasis/health_professionals/index.html#dx ) |
| soil transmitted helminths | detection of helminth/ eggs in stool samples by microscopy (or) PCR |  |  |
| Schistosomiasis | Identification of parasite eggs (stool for *S. mansoni* or S. japonicum eggs and urine for *S. haematobium* eggs). Antibodies and/or antigens detection in blood |  | <https://www.who.int/news-room/fact-sheets/detail/schistosomiasis> |
| Mycetoma | The diagnosis of mycetoma is based on clinical presentation and identification of the causative organisms which can be detected by directly examining the grains that are discharged by the sinuses. The samples can be obtained by Fine Needle Aspiration (FNA) or surgical biopsy. Imaging (Ultrasound/ MRI) can also be used. |  | <https://www.cdc.gov/fungal/diseases/mycetoma/health-professionals.html> |
| Note:  1. We defined cases of corresponding infections/ diseases based on one of the diagnostic tests mentioned above (either from low or high-risk category)  2. If the article does not mention detail method of diagnosis, it is regarded as "Not Clear" | | | |
