# Supplementary material for: A systematic review of neglected tropical diseases (NTDs) in Myanmar
Source: PLoS Negl Trop Dis. 2023 Nov 1;17(11):e0011706. doi: 10.1371/journal.pntd.0011706 (PMC10619876; doi:10.1371/journal.pntd.0011706)
Supplement: S3 Table — (DOCX) [file pntd.0011706.s004.docx]

**Supplementary Table 3. Reported NTDs stratified by aetiology and age groups.**

|  | **Child**  **(< 12 years)** | **Adolescents and adult**  **(≥ 12 years)** | **Both age groups** | **Age not specified** | **Total** |
| --- | --- | --- | --- | --- | --- |
| **Bacterial NTDs (n=320)** |  |  |  |  |  |
| *E. coli* enteritis | 36 | 3 | 8 | 3 | 50 |
| Cholera | 13 | 8 | 10 | 12 | 43 |
| Salmonellosis | 13 | 22 | 4 | 8 | 47 |
| Shigellosis | 18 | 5 | 6 | 4 | 33 |
| Leprosy | 2 | 14 | 14 | 29 | 59 |
| Melioidosis | 0 | 25 | 0 | 7 | 32 |
| Rickettsioses | 0 | 13 | 3 | 3 | 19 |
| Leptospirosis | 1 | 10 | 3 | 6 | 20 |
| Trachoma | 0 | 1 | 3 | 7 | 11 |
| Syphilis | 0 | 3 | 0 | 1 | 4 |
| Yaws | 0 | 0 | 1 | 1 | 2 |
| **Sub-total** | 83 | 104 | 52 | 81 | 320 |
|  |  |  |  |  |  |
| **Viral NTDs (n=244)** |  |  |  |  |  |
| Dengue | 109 | 35 | 15 | 25 | 184 |
| Chikungunya | 13 | 7 | 4 | 6 | 30 |
| JEV infection | 15 | 2 | 3 | 2 | 22 |
| Rabies | 1 | 1 | 1 | 2 | 5 |
| Zika virus disease | 0 | 2 | 1 | 0 | 3 |
| **Sub-total** | 138 | 47 | 24 | 35 | 244 |
|  |  |  |  |  |  |
| **Protozoal NTDs (n=65)** |  |  |  |  |  |
| Amoebiasis | 12 | 10 | 5 | 10 | 37 |
| Giardiasis | 12 | 6 | 4 | 2 | 23 |
| Leishmaniasis | 0 | 3 | 0 | 1 | 4 |
| **Sub-total** | 24 | 19 | 9 | 13 | 65 |
|  |  |  |  |  |  |
| **Helminth (n=312)** |  |  |  |  |  |
| Ascariasis | 46 | 18 | 15 | 9 | 88 |
| Hookworm disease | 20 | 12 | 13 | 5 | 50 |
| Strongyloidiasis | 6 | 20 | 4 | 2 | 32 |
| Microfilariasis | 0 | 21 | 13 | 3 | 37 |
| Trichuriasis | 35 | 12 | 12 | 6 | 65 |
| Taeniasis/ Cysticercosis | 3 | 6 | 2 | 0 | 11 |
| Trematodes | 1 | 3 | 1 | 0 | 5 |
| Soil transmitted helminths | 17 | 2 | 5 | 0 | 24 |
| **Sub-total** | 128 | 94 | 65 | 25 | 312 |
|  |  |  |  |  |  |
| **Fungus (n=4)** |  |  |  |  |  |
| Mycetoma | 0 | 4 | 0 | 0 | 4 |
| **Sub-total** | 0 | 4 | 0 | 0 | 4 |
| Ectoparasite (n=1) |  |  |  |  |  |
| Myiasis | 0 | 1 | 0 | 0 | 1 |
| **Sub-total** |  |  |  |  | 1 |
| **Total** | 373 | 269 | 150 | 154 | 946 |
