# Supplementary material for: A systematic review of neglected tropical diseases (NTDs) in Myanmar
Source: PLoS Negl Trop Dis. 2023 Nov 1;17(11):e0011706. doi: 10.1371/journal.pntd.0011706 (PMC10619876; doi:10.1371/journal.pntd.0011706)
Supplement: S4 Table — (DOCX) [file pntd.0011706.s005.docx]

**Supplementary Table 4. Geographic location of reported NTDs in included articles**

| **States/ Regions** | **No of reported NTDs** | | | | | | **No of articles** |
| --- | --- | --- | --- | --- | --- | --- | --- |
|  | **Bacterial NTDs** | **Viral NTDs** | **Protozoal NTDs** | **Helminth NTDs** | **Fungal NTDs** | **Ectoparasitic NTDs** |  |
| Yangon | 181 | 125 | 33 | 126 | 2 | 1 | 302 |
| Mandalay | 20 | 47 | 4 | 20 |  |  | 67 |
| Magway | 5 | 2 | 2 | 16 |  |  | 14 |
| Ayeyarwaddy | 2 | 3 | 0 | 7 |  |  | 8 |
| Sagaing | 3 | 0 | 0 | 0 |  |  | 3 |
| Bago | 9 | 1 | 0 | 18 |  |  | 15 |
| Tanintharyi | 1 | 1 | 1 | 0 |  |  | 2 |
| Naypyidaw | 3 | 1 | 0 | 7 |  |  | 7 |
| Mon | 1 | 2 | 0 | 2 |  |  | 4 |
| Kayin | 0 | 1 | 0 | 0 |  |  | 1 |
| Kayar | 0 | 0 | 0 | 3 |  |  | 1 |
| Kachin | 0 | 0 | 0 | 3 |  |  | 5 |
| Shan | 0 | 2 | 2 | 3 |  |  | 6 |
| Rakhine | 4 | 3 | 2 | 0 |  |  | 7 |
| Multiple regions | 27 | 12 | 0 | 27 |  |  | 50 |
| Nationwide | 4 | 8 | 0 | 2 |  |  | 13 |
| Thailand-Myanmar border | 20 | 4 | 4 | 29 |  |  | 29 |
| Other countries | 15 | 9 | 9 | 38 | 2 |  | 51 |
| Not specified | 45 | 25 | 8 | 30 |  |  | 87 |
| **Total** | 339 | 246 | 67 | 331 |  |  | 672 |
