# Supplementary figures and images for: A systematic review of neglected tropical diseases (NTDs) in Myanmar
Source: PLoS Negl Trop Dis. 2023 Nov 1;17(11):e0011706. doi: 10.1371/journal.pntd.0011706 (PMC10619876; doi:10.1371/journal.pntd.0011706)

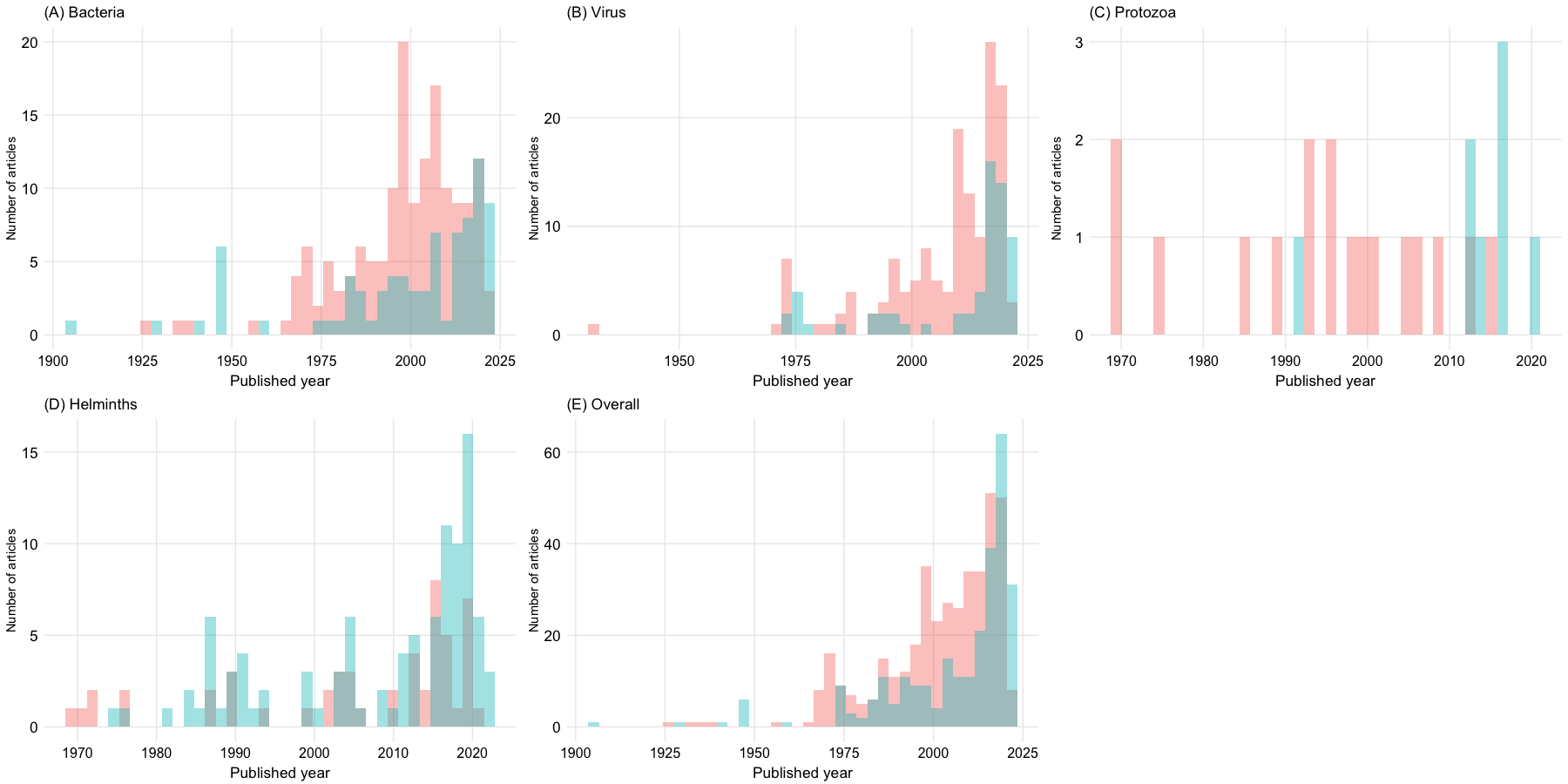

Supplement: S1 Fig — For clarity, four articles reporting mycoses and one reporting myiasis are not shown in the figure. (TIF) [file pntd.0011706.s007.tif]
